# Inhibitors of endopeptidase and angiotensin-converting enzyme lead to an amplification of the morphological changes and an upregulation of the substance P system in a muscle overuse model

**DOI:** 10.1186/1471-2474-15-126

**Published:** 2014-04-11

**Authors:** Yafeng Song, Per S Stål, Ji-Guo Yu, Ronny Lorentzon, Clas Backman, Sture Forsgren

**Affiliations:** 1 Department of Integrative Medical Biology, Section for Anatomy, Umeå University, 901 87 Umeå, Sweden; 2 Department of Surgical and Perioperative Sciences, Sports Medicine, Umeå University, Umeå, Sweden; 3 Department of Surgical and Perioperative Sciences, Hand Surgery, Umeå University, Umeå, Sweden

**Keywords:** Muscle, Exercise, Overuse, Myositis, Tachykinin, Substance P

## Abstract

**Background:**

We have previously observed, in studies on an experimental overuse model, that the tachykinin system may be involved in the processes of muscle inflammation (myositis) and other muscle tissue alterations. To further evaluate the significance of tachykinins in these processes, we have used inhibitors of neutral endopeptidase (NEP) and angiotensin-converting enzyme (ACE), substances which are known to terminate the activity of various endogenously produced substances, including tachykinins.

**Methods:**

Injections of inhibitors of NEP and ACE, as well as the tachykinin substance P (SP), were given locally outside the tendon of the triceps surae muscle of rabbits subjected to marked overuse of this muscle. A control group was given NaCl injections. Evaluations were made at 1 week, a timepoint of overuse when only mild inflammation and limited changes in the muscle structure are noted in animals not treated with inhibitors. Both the soleus and gastrocnemius muscles were examined morphologically and with immunohistochemistry and enzyme immunoassay (EIA).

**Results:**

A pronounced inflammation (myositis) and changes in the muscle fiber morphology, including muscle fiber necrosis, occurred in the overused muscles of animals given NEP and ACE inhibitors. The morphological changes were clearly more prominent than for animals subjected to overuse and NaCl injections (NaCl group). A marked SP-like expression, as well as a marked expression of the neurokinin-1 receptor (NK-1R) was found in the affected muscle tissue in response to injections of NEP and ACE inhibitors. The concentration of SP in the muscles was also higher than that for the NaCl group.

**Conclusions:**

The observations show that the local injections of NEP and ACE inhibitors led to marked SP-like and NK-1R immunoreactions, increased SP concentrations, and an amplification of the morphological changes in the tissue. The injections of the inhibitors thus led to a more marked myositis process and an upregulation of the SP system. Endogenously produced substances, out of which the tachykinins conform to one substance family, may play a role in mediating effects in the tissue in a muscle that is subjected to pronounced overuse.

## Background

A pronounced muscle inflammation (myositis) and a derangement of the structure of muscle tissue occur in inflammatory myopathies [1,2]. Muscle inflammation and alterations of muscle structure also occur in response to muscle overuse after heavy exercise and in situations with repetitive strain injury [3]. We have recently studied the development of myositis, as well as muscle tissue derangement, in response to muscle overuse in the triceps surae muscle of the rabbit [4]. Using this overuse model, we have observed that there is an upregulation in the expression of tachykinin [substance P (SP)] and the SP-preferred receptor [the neurokinin-1 receptor (NK-1R)] in muscle areas showing inflammation/tissue derangement [5,6]. All these features were clearly noticeable after 3-6 weeks of experimental overuse and to a small extent after an experimental period of 1 week. The observations suggest that tachykinins are involved in the myositis/derangement processes.

SP is the most well-known peptide in the tachykinin family. It is found in the C fiber population of primary afferent neurons and is released from their central and peripheral endings [7,8]. SP is also present in non-neuronal cells such as white blood cells [9]. SP has marked pro-inflammatory properties [10-12], as evidenced by participation in neurogenic inflammation [13,14] and in the processes of extravasation and accumulation of inflammatory cells [15]. Increased SP effects are considered to lead to an up-regulation of NK-1R expression, consistent with an activation of the NK-1 R [16,17].

Exogenous administration of SP via injections or via topical treatment has been tested experimentally and has been shown to have effects in a large number of inflammatory/destructive events, as well as in repair and wound-healing processes [18-20]. These include repair processes in models of Achilles tendon rupture [21,22]. Local administration of SP outside the Achilles tendon has been found to accelerate hypercellularity and angiogenesis within this tendon in an experimental tendinopathy-like situation [23].

The activity of tachykinins, such as SP, and certain other peptide mediators is terminated by enzymatic degradation and by receptor inactivation. The most important enzymes are neutral endopeptidase (NEP), which degrades SP in the extracellular space [24-26], and angiotensin-converting enzyme (ACE) [27]. Local injections of SP and/or endopeptidase inhibitor are reported to have a booster effect on endogenous SP production [22]. The present study was undertaken in order to further determine the role for tachykinin involvement in muscle derangement and inflammation processes. We have used the rabbit overuse model to study this. We performed the combined exercise/electrical stimulation experiment for 1 week and in order to obtain pronounced effects of endogenously produced substances, we gave local treatments with ACE- and NEP-inhibitors. SP was also given. It was hypothetized that the exogenous treatments with ACE- and NEP-inhibitors, as well as SP, would lead to pronounced activation of the NK-1R and an increased inflammation process and tissue derangement.

## Methods

### Animals

In the experiments, female New Zealand white rabbits with a weight of approximately 4 kg (age ranging from 6 to 9 months) were utilized. In total, the investigation included evaluations of 16 animals. All the animals were subjected to an exercise protocol leading to marked overuse of the triceps surae muscle. The muscle overuse was combined with injection treatment (see below).

### Exercise procedure

The animals were exposed to an exercise procedure that was performed according to previously described procedures [28], with some modifications [4-6,23]. The rabbits were kept under anaesthesia, induced by i.m. injections of diazepam (5 mg/ml; 0.2 ml/kg) and fentanylfluanison (0.2-0.3 ml/kg), throughout the experiment. Fentanylfluanison (0.1 ml/kg) was injected each 30-45 min during the experiment in order to maintain the anaesthesia. The experiments lasted for 2 hours. For analgesia, buprenorphine (0.01-0.05 mg/kg) was given s.c. after each experimental session. The experiment was repeated every second day for one week (4 exercise sessions in total).

In order to achieve passive repetitive flexions and extensions of the right ankle joint, an apparatus (“kicking machine”) was used. A pneumatic piston attached to the foot produced the movements. An active contraction was furthermore induced by electrical stimulation via surface electrodes (Pediatric electrode 40 426A, Hewlett Packard, Andover, MA, USA) placed 2 cm apart over the triceps surae muscle of the right leg. This active contraction occurred during the plantar flexion phase. The stimulation was synchronized with the plantar flexion movement of the piston by a microswitch, which trigged the stimulator unit (Disa stimulator Type 14E, Disa Elektronik A/S, Herlev, Denmark). An impulse of 0.2 ms duration was delivered 85 ms after the initiation of the plantar flexion at an amplitude of 35-50 V. The movement frequency was 150 movements per minute. The left leg was not attached to the kicking machine and the pelvis was strapped down. In-between the experimental periods, the animals were kept in ordinary cages allowing good freedom of movement. For further details about the apparatus and the exercise protocol, see [4-6,23].

### Injection treatments

Injections were given in the loose connective tissue outside the Achilles tendon, i.e. in the paratenon region, of the right leg. The injections were given directly after each of the exercise regimens. The substances injected were Captopril (C) (c4042; Sigma), DL-Thiorphan (Th) (T 6031; Sigma), substance P (SP) (S6883; Sigma) and NaCl. Captopril is an angiotensin-converting enzyme inhibitor (ACE inhibitor) and DL-Thiorphan inhibits the activity of neutral endopeptidase (NEP). NEP is also called enkephalinase and neprilysin or common acute lymphoblastic leukemia antigen or CD10 [29].

Injections were performed as follows: a) Substance P (10^-8^ μmol/ml) and Captopril (30 μmol/kg) both in distilled water (volume: 1 ml) + Dl-Thiorphan (500 μg/ml; 0.02 ml) (5 animals), b) Captopril (30 μmol/kg, dissolved in distilled water, volume 1 ml) + DL-Thiorphan (500 μg/ml, 0.02 ml) (6 animals), c) NaCl (volume: 1 ml) (5 animals). The groups are further referred to as the SP + C + Th group, the C + Th group and the NaCl group.

### Collection of muscle samples, fixation and sectioning

The animals were sacrificed, using an overdose of Pentobarbital, the day after the last exercise. The triceps surae muscles were dissected out. Directly thereafter the muscle samples were transported on ice to the laboratory. The distal parts of the soleus and gastrocnemius muscles (5-8 × 10 mm) were dissected out. Specimens from all samples were collected and processed in three ways: Immersion fixed, directly embedded/frozen or directly frozen in liquid nitrogen. For all types of specimens, specimens from all the various muscles were obtained. Thus, for both the soleus and gastrocnemius muscles, 5-6 specimens were generated for each experimental condition.

For fixation, specimens were fixed by immersion overnight at 4°C in an ice-cold solution of 4% formaldehyde in 0.1 M phosphate buffer (pH 7.0). These specimens were thereafter thoroughly washed in Tyrode’s solution containing 10% sucrose at 4°C overnight, mounted on thin cardboard in OCT embedding medium (Miles Laboratories, Naperville, Ill.), and frozen in propane chilled with liquid nitrogen. Other specimens were, as described above, directly embedded and frozen. Both types of specimens were stored at -80°C and from both types, series of 7-8 μm thin sections were cut using a cryostat. The sections were mounted on slides precoated with chrome-alum gelatine and were then processed for morphology (chemically unfixed samples) or immunohistochemistry and morphology (chemically fixed samples). Other specimens weighing approximately 30 mg were directly frozen in liquid nitrogen. They were further used for EIA analyses.

From one of the experimental groups (the SP + C + Th group) specimens, conforming to all three types of specimens as described above, were collected from both the right and left legs.

### Processing for morphology (haematoxylin & eosin)

Sections in the series were stained in Harris Haematoxylin solution for 2 min. They were then rinsed in distilled water, dipped in 0.1% acetic acid for a few seconds, followed by washing in running water. Counterstaining was achieved by immersion in eosin for 1 min. The sections were dehydrated in ethanol and mounted in Permount.

### Morphological semiquantitative evaluations

Evaluations with respect to the magnitude of morphological changes were made. The evaluations were based on the haematoxylin & eosin (H&E) stainings. The evaluations were made blinded.

The degree of inflammatory infiltrates in the tissue and the degree of presence of abnormal connective tissue areas, necrotic muscle fibers and variations in muscle fiber sizes as well as the degree of occurrence of internal nuclei were all assessed. An overall estimation for all these parameters was performed. The evaluations included an overall estimation of the entire sections from both the chemically unfixed and fixed specimens of each muscle.

The following pattern of scoring was used: 0 = no changes; morphology resembling that seen in normal muscle tissue, 1 = mild degree of changes, 2 = moderate degree of changes, 3 = marked changes, 4 = very marked changes. The scoring was relative, i.e. the magnitude of changes in one muscle was related to those in others. The mean value for scores for the different groups was then calculated. The significance level was set at the 0.05 level. The scoring is a simplified scoring method in relation to the one used in our previous morphological study on the effects of 1,3 and 6 weeks of experiment [4]. Based on the experience we obtained from that study, we consider that the scoring procedures that were presently used are reliable. Thus, the degree of occurrence of the different abnormal features analyzed parallelled each other.

### Immunohistochemistry

#### Staining procedures for demonstration of substance P (SP) and NK-1R

Two antibodies for staining for demonstration of SP were used. One of the SP antibodies and the NK-1 R antibody were goat polyclonal antibodies (see below). The staining procedures for these antibodies conformed to those previously described [5,6]. This included the use of 5% normal donkey serum (code no: 017-000-121, Jackson ImmunoResearch Lab. Inc., PA) as normal serum and FITC-conjugated AffiniPure donkey antigoat IgG (705-095-147; Jackson ImmunoResearch Lab Inc, dilution 1:100) as secondary antiserum. Mounting was performed in Vectashield Mounting Medium (H-1000) (Vector Laboratories, Burlingame, CA) and examination was carried out in a Zeiss Axioscope 2 plus microscope equipped with an Olympus DP70 digital camera.

Staining using a monoclonal SP antibody was also performed. In this case, an initial incubation with Triton X-100 was done, followed by incubation in 5% normal donkey serum and incubation with the primary antibody, diluted in PBS with BSA, in a humid environment. Incubation was performed for 60 min at 37°C. Another incubation in normal donkey serum followed, after which the sections were incubated with secondary antiserum. As secondary antiserum, tetramethylrhodamine isothiocyanate (TRITC)-conjugated AffiniPure donkey anti-rat IgG (712-025-150) (Jackson ImmunoResearch, PA), diluted 1:40 in PBS supplemented with 0.1% BSA, was used. Incubation with secondary antiserum proceded for 30 min at 37°C. The procedures for mounting and examination were as described above.

As an alternative procedure for demonstration of SP and NK-1R immunoreactions, sections were initially pretreated with acid potassium permanganate for 2 min, a procedure found to enhance specific immunofluorescence reactions [30]. After this pretreatment, the procedures described above followed. Both procedures (with or without acid potassium permanganate treatment) gave in principle similar results. There were, however, some differences in staining intensities and levels of background reactions, why it was useful to analyse sections by using both types of procedures.

#### Stainings for demonstration of desmin and CD31

Immunostaining for desmin was used in order to verify the occurrence of muscle fiber necrosis since it is well-known that necrotic muscle fibers show no or negligible desmin immunoreactivity [31,32]. CD31 is a well-known marker of endothelia of blood vessels.

The CD31 staining was performed using visualization with FITC-conjugated donkey anti-mouse IgG (715-095-15) (Jackson ImmunoResearch), dilution used 1:100, and as normal serum, normal donkey serum with PBS in BSA was utilized. For the evaluation of desmin reaction patterns, double staining desmin/NK-1R was made. FITC-conjugated AffiniPure donkey anti-goat IgG (705-095-147) was used as secondary antibody for visualization of NK-1R and 5% normal donkey serum in PBS was used as normal serum. Concerning desmin immunodetection, rabbit anti-mouse immunolobulins/TRITC (R0276) (Dako, Denmark) was used as secondary antiserum, 1:40 dilution being used, and 5% normal rabbit serum in PBS with BSA as normal serum. Mounting was made in Vectashield Mounting Medium (H-1000).

#### Antibodies and control stainings

##### SP antibodies

An affinity purified goat polyclonal SP antibody was utlized. It was obtained from Santa Cruz Biotechnology (code: sc-14104) and was used at a dilution of 1:25-1:50. This antibody is raised against a peptide mapping within an internal region of preprotachykinin 1 of human origin and is recommended for detection of mature SP and all isoforms of the protachykinin 1 precursor of various species [mouse, rat and human origin]. According to “Human Protein Reference Database” and “BLAST-Basic Local Alignment Search Tool”, the protein sequence of rabbit protachykinin 1 shows at least 85% identity with human protachykinin 1.

A rat monoclonal SP antibody was also used. It was obtained from Biogenesis, Poole, UK (code: 8450-0505). It was used at a dilution of 1:50-1:100. The antibody is reported to recognize the COOH terminal end of SP and has been previously used for immunohistochemical detection of SP in experimental animals and man [5,33].

Control stainings included the use of SP blocking substance (code: sc14104P; Santa Cruz) (20-50 μg/ml antiserum) and SP peptide (full length SP peptide; S6883; Sigma) (50 μg/ml antiserum). Ordinary stainings with the SP antisera were performed in parallel. Other control stainings conformed to stainings when the primary antibodies were excluded (buffer instead of the antibody).

##### NK-1R antibody

A NK-1R antibody produced in goats was used (code: sc-5220, Santa Cruz). It is an affinity purified polyclonal antibody raised against a peptide mapping within an internal region of NK-1R of human origin. It was used at a dilution of 1:50-1:100 in 0.1% PBS. It has been previously used in studies on immunoexpression patterns in rabbit muscle tissue [6]. Control stainings included the use of NK-1R blocking substance (sc-5220P) (50 μg/ml antiserum). Ordinary stainings for NK-1R were made in parallel. As was the case concerning stainings for SP, other control stainings conformed to stainings when the primary antibody was excluded.

##### CD31 and desmin antibodies

Mouse monoclonal antibodies against CD31 and desmin were used. The CD31 antibody was obtained from Dako, Glostrup, Denmark (code: M0823) and was used at a dilution of 1:100. It is a marker of the endothelial cells of blood vessel walls and has previously been used with reliable results in our laboratory [23]. The desmin antibody was also obtained from Dako, Glostrup, Denmark (code: Ab D33) and was utilized at a dilution of 1:100. It is by the supplier reported to be specific for desmin and to not show reactivity with other types of intermediate filaments. This antibody has been evaluated in previous studies [4].

### Processing for EIA

#### Tissue homogenization

After being frozen in liquid nitrogen (see above), the samples were homogenised, by mechanical homogenization using a Precellys 24 tissue homogenizer (Bertin Technologies, Saint Quentin en Yvelines, Cedex, France), in a 100 mM Tris-HCl buffer, pH 7.0, containing 1 M NaCl, 2% BSA, 4 mM EDTA, 0.2% Triton X-100 (pH 7.0), 0.02% Na-azide and the protease inhibitors Pepstatin A (0.1 μg/ml), Aprotinin (5 μg/ml), Antipain (0.5 μg/ml), Benzamidin (167 μg/ml) and PMSF (5.2 μg/ml). All the protease inhibitors were purchased from Sigma, Germany. Tissue and buffer were mixed in a 1:20 ratio. The procedure was performed on ice. Directly after the homogenisation procedure, the tissue samples were centrifuged in +4°C, 13 000 g, for 15 min. The supernatant was then transferred to a new Eppendorf tube and stored at -80°C.

#### EIA procedure

The concentration of SP in the muscle samples was evaluated using commercially available enzyme immunoassay SP kits (Phoenix Pharmaceuticals, Burlingame, CA, USA). The sequence of the peptide detected conforms to the full-length of SP. 100% cross-reactivity is also reported for SP 2-11 – SP 5-11. Less than 0.01% cross-reactivity is reported for SP 7-11, neuropeptide K and neurokinin A and 0% for neurokinin B. The assays were performed in accordance with the instructions from the manufacturer. In order to obtain comparable results between different plates, reference samples were included in the various analyses. The levels of SP were normalized to the weights of the tissue samples, i.e. the values are expressed as pg/mg tissue.

### Statistics

All data are expressed as mean and standard deviations (SD) are given. A one way ANOVA in combination with post hoc comparisons by use of Least Square Difference (LSD) was used for analysis of differences between the different groups. The paired *t* test was used for the evaluation of possible differencees in SP concentration between the experimental and non-experimental sides (concerning SP + C + Th group). The normality for the data for each group was examined and the distribution was found to be normal or approximately normal. All the statistical analysis was performed by software SPSS (PASW Statistics 20). A p-value < 0.05 was considered to be significant.

### Ethics

The study protocol was approved by the local ethical committee at Umeå University (A34/07, A95/07). A licensed breeder had bred all the animals for the sole purpose of being used in animal experiments. All efforts were made to minimize animal suffering.

## Results

### Morphology

#### Microscopic observations

**Figure 1:**
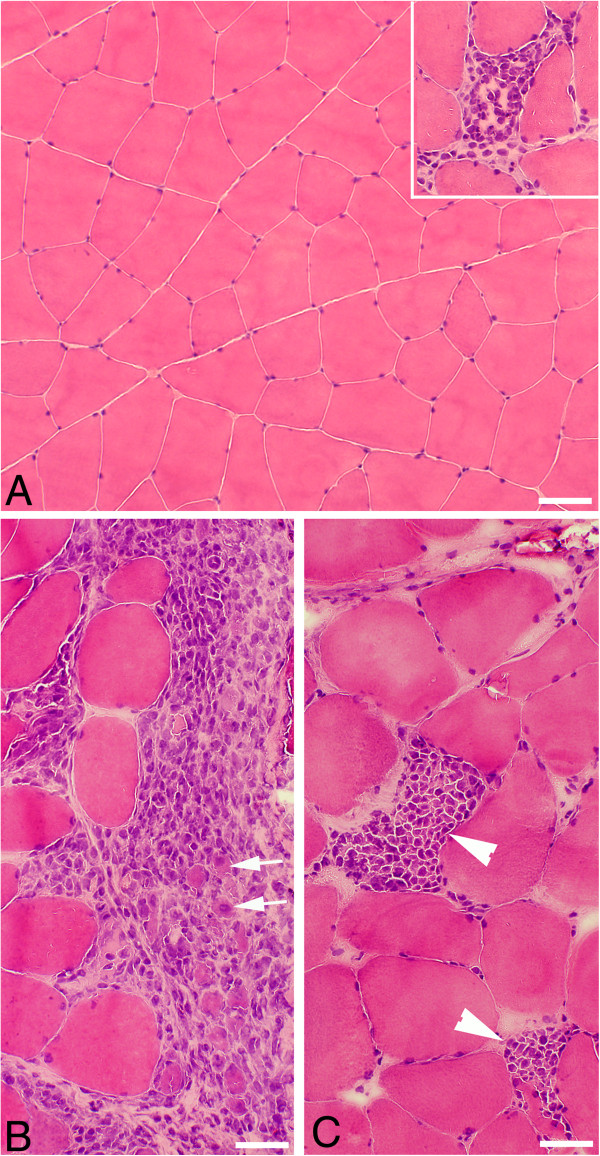
**Muscle tissue of the gastrocnemius (A) and soleus (inset, A) muscles.** The animals had been subjected to muscle overuse coupled with local injections with NaCl. The muscle tissue shows a normal organization, and very occasional presence of necrotic muscle fibers (middle in inset). Bar = 50 μm. Muscle tissue of gastrocnemius muscles from two animals subjected to muscle overuse coupled with local injections with SP + C + Th; non-exercised side **(B)**, exercised side **(C)**. There is a variability in muscle fiber appearance, a very marked inflammatory infiltrate (to the right, B) and necrotic muscle fibers (arrowheads, C). There is a presence of very small muscle fibers in the inflammatory infiltrate (arrows, B). Bars = 50 μm.

**Figure 2:**
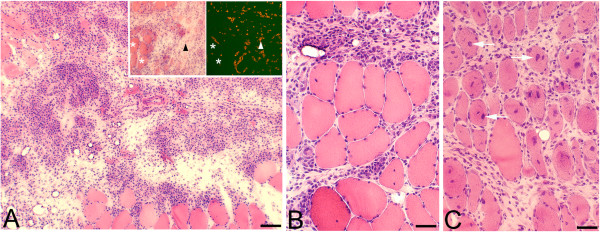
**Tissues of soleus muscle of animals subjected to muscle overuse coupled with injections of C + Th.** There is a very marked inflammatory infiltrate in **(A)**, most of the area shown being occupied by infiltrated white blood cells and loose connective tissue. There is an infiltration of white blood cells and a marked presence of connective tissue in-between muscle fibers in **(B)** and **(C)**. The muscle fibers in **(C)** show frequently internal nuclei (arrows). In the inset in **(A)**, two parallel sections stained with H&E (figure to the left) and stained for demonstration of CD31 (figure to the right), are shown. There are numerous CD31 reactions in the inflammatory/connective tissue area (triangles), reactions which based on what is known for CD31 are related to endothelia of blood vessels. Asterisks at muscle fibers. Bars = 100 μm **(A)**, 50 μm **(B, C)**.

Extensive morphological changes occurred for both the soleus and gastrocnemius muscles in the animals given local injections with C + Th or SP + C + Th (Figures 1 and 2). The changes were particularly marked in the C + Th group. In the SP + C + Th animals, for which both the experimental (exercised) and contralateral sides were examined, the morphological changes appeared similar in both sides.

**Figure 3:**
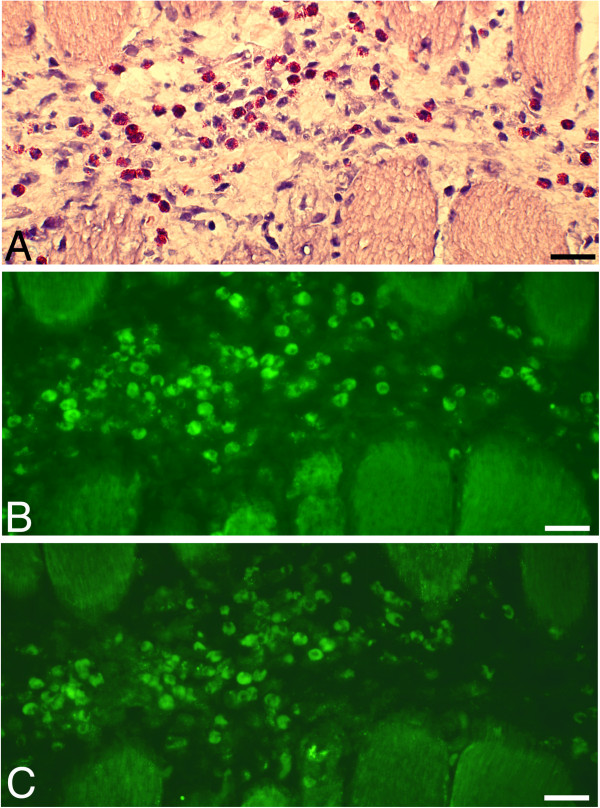
**A series of sections of a soleus muscle specimen of an animal subjected to muscle overuse and injections with C + Th.** The sections were stained with H&E **(A)**, and processed for SP (antibody sc-14104) **(B)** and NK-1R **(C)**. There are numerous white blood cells in a connective tissue area located in-between muscle fibers. There is presence of SP-like and NK-1R immunoreactions in these cells. A large number of the cells in this area are eosinophils. Bars = 25 μm.

The morphological changes corresponded particularly to pronounced inflammatory cell infiltrations, occurrence of necrotic muscle fibers (i.e. fibers that were markedly infiltrated by inflammatory cells) and the frequent presence of internal nuclei in the muscle fibers as well as an abnormal presence of wide areas with loose connective tissue (Figures 1, 2 and 3A). Muscle fibers with variable sizes were encountered in the inflammatory areas (Figure 1B). The morphological changes were not randomly occurring in the specimens but were mainly concentrated to certain areas (“myositis areas”).

When compared with the muscles of the NaCl group, it was noted that the morphological changes were clearly more pronounced for the muscles of the C + Th and SP + C + Th groups (c.f. Figure 1A). Morphological alterations such as occurrence of necrotic fibers were only sometimes observed in the NaCl group (Figure 1A).

#### Semiquantitative evaluations

Semiquantitative evaluations of the magnitude of the occurring morphological changes were made (see Methods).

a) Soleus muscle

There was a clearly higher mean value concerning the degree of morphologic changes in the C + Th group as compared with the NaCI group (3.8 vs. 1.5, p = 0.0002). A high mean value was also noted for the SP + C + Th group (non-experimental side) as compared to the NaCI group (3.0 vs. 1.5, p = 0.061). There was a higher mean value for the muscle of the experimental side in the SP + C + Th group as compared to that of the NaCI group, but the difference was not statistically significant (2.3 vs. 1.5, respectively, p = 0.236). The mean values are shown in Table 1.

b) Gastrocnemius muscle

There were higher mean scores for the SP + C + Th group (both experimental and non-experimental sides) and the C + Th group as compared to the NaCI group. However, the differences were not statistically significant. The mean values for all the various groups are shown in Table 1.

**Table 1:** Mean values of the scoring of morphological changes and the mean values of SP concentations as measured by EIA in the soleus (SOL) and gastrocnemius (GM) muscles for the NaCI, SP + C + Th and C + Th groups are shown

	**Scores for morphology**		**SP concentration (pg/mg)**	
**Group**	**SOL**	**GM**	**SOL**	**GM**
NaCI (n = 5)	1.5 ± 1.1	1.2 ± 0.4	240 ± 50	353 ± 147
SP + C + Th (exp) (n = 5)	2.3 ± 0.8	2.0 ± 1.3	351 ± 75 (363)	511 ± 134^#^
SP + C + Th (non-exp) (n = 5)	3.0 ± 1.1	2.1 ± 0.7	295 ± 67	450 ± 55
C + Th (n = 6)	3.8 ± 0.6^#^	2.2 ± 0.5	509 ± 160^#▲□^ (422)	531 ± 104^#^

For the SP + C + Th group, samples were obtained from both the experimental (exp) and non-experimental (non-exp) sides. For the other groups, the samples were solely from the experimental side. Values within brackets (concerning SP concentration, SOL) are the mean values if samples for which CV was above 15% were excluded (CV was above 15% concerning values for in total 3 samples out of the 42 samples analyzed).

One way ANOVA in combination with post hoc comparisons was applied for the analysis of differences between the groups concerning SP concentration. Significance level was set at 0.05. Symbols concerning occurrence of statistical significances are shown. ^#^Significant difference as compared to NaCI group. ^▲^Significant difference between the C + Th group and the SP + C + Th group (experimental side), ^□^significant difference between the C + Th group and the SP + C + Th group (non-experimental side).

### Immunohistochemical observations

#### C + Th and SP + C + Th groups

##### SP-like immunoreactions

The two SP antibodies used were directed against different epitopes (see Materials & Methods). As will be described below, there was a difference in the reaction pattern for the two antibodies. We further on use the terminology “SP-like” immunoreactivity to describe the reactions obtained using the two antibodies. The terminology “tachykinin-like” was used in a previous study [5].

Marked SP-like immunoreactions were observed in myositis areas and areas adjacent to these in the C + Th group and both sides of the SP + C + Th group. The magnitude of immunoreactions was especially high in the C + Th group.

**Figure 4:**
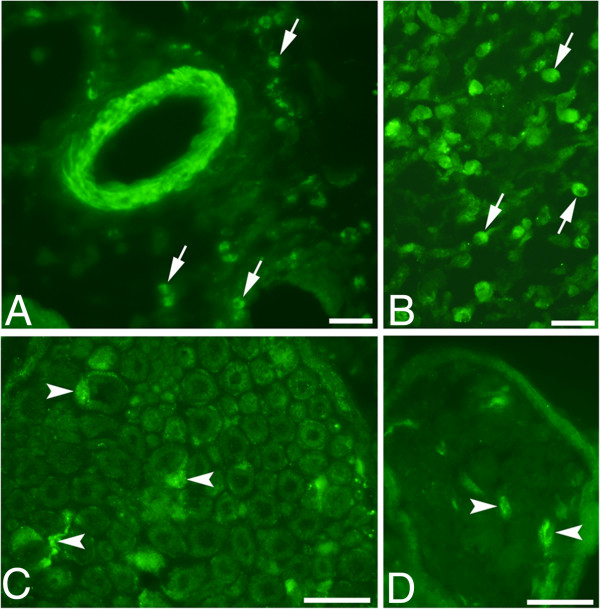
**Sections of soleus muscle specimens; C + Th group (A-C) and SP + C + Th group, non-exercised side (D).** The sections were stained for SP (antibody sc-14104) **(A,C)** and NK-1R **(B,D)**. In **(A)**, an arteriole with marked tachykinin-like/(SP-Like) immunoreactions in its wall is seen (middle) and the region shown in **(B)** conforms to a region with marked infiltration of white blood cells. SP-like/ immunoreactions **(A)** and NK-1R immunoreactions **(B)** are seen in white blood cells (arrows). Parts of nerve fascicles are seen in **(C)** and **(D)**. Immunoreactions are seen in these in both **(C)** and **(D)** (arrows). Bars = 25 μm.

**Figure 5:**
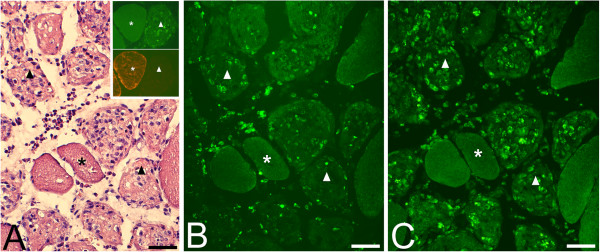
**A series of sections of a soleus muscle specimen from the SP + C + Th group (non-exercised side).** The sections were stained with H&E **(A)**, and processed for SP (antibody sc-14104) **(B)** and NK-1R **(C)**. Muscle fibers that largely are normal in appearance (asterisk) and muscle fibers that are more or less filled with white blood cells (triangles) (necrotic muscle fibers) are seen. Marked NK-1R and to some extent also SP-like immunoreactions are abservable in the white blood cells that have infiltrated into the necrotic muscle fibers. There are also NK-1R and SP-like immunoreactions in white blood cells located in the connective tissue spaces that separate the muscle fibers. Note that there is also some unspecific autofluoresence reactions in muscle fibers. In the inset in **(A)**, the results of double-staining for NK-1R (above) and desmin (below) are demonstrated. One muscle fiber shows marked NK-1 R immunoreaction but no desmin immunolabelling (triangle), whist others only show unspecific background raction after NK-1 R staining but specific desmin immunolabelling (asterisks). The former fiber can based on desmin reaction pattern be interpreted to be a necrotic muscle fiber. Bars = 50 μm.

**Figure 6:**
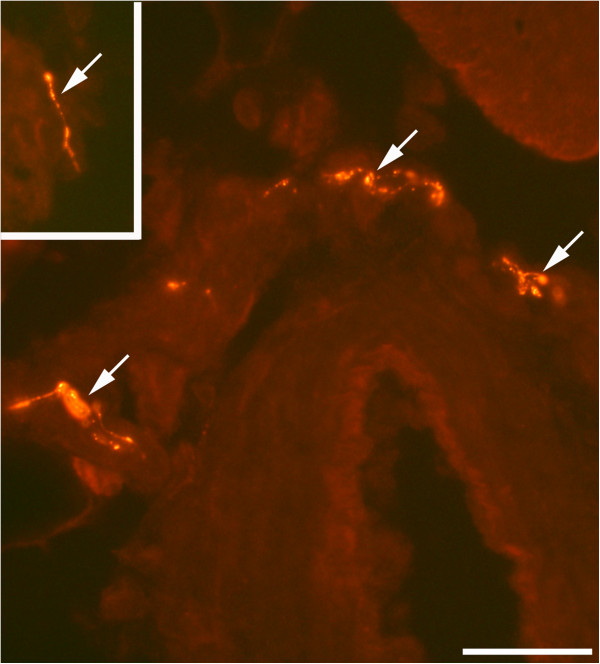
**SP immunoreactivity features (staining with antibody 8450-0505).** The specimen was from the SP + C + Th group; non-exercised side. There are frequent fine varicose nerve fibers exhibiting SP-like immunoreaction outside the wall of an arteriole (arrows, main figure) and in a connective tissue space (arrow, inset). Bar = 25 μm.

The SP-like immunoreactions were seen in the infiltrating white blood cells, blood vessel walls and nerve profiles. Reactions in white blood cells occurred for such cells that were dispersed in connective tissue areas (Figures 3B and 4A) as well as for such cells that were coalesced into necrotic muscle fibers (Figure 5B). Blood vessels showing SP-like immunoreactions in their walls were mainly seen in myositis areas and areas that were adjacent to these (Figure 4A). The nerve fascicles located in these areas showed partially SP-like immunoreactions (Figure 4C). Frequent varicose nerve profiles showing SP-like immunoreaction were seen in association with blood vessel walls and in the connective tissue in myositis areas (Figure 6).

Specific immunoreactions were obtained for nerve fibers and white blood cells using both the polyclonal and monoclonal antibodies, whilst specific immunoreactions in the blood vessel walls were only obtained with the polyclonal antiserum. The polyclonal antibody thus depicts a broader tachykinin spectrum, the antibody detecting all isoforms of the protachykinin 1 precursor.

##### Immunoexpression of NK-1 R

Very marked NK-1R immunoreactions were noted for the C + Th group and for both sides of the SP + C + Th group. As was the case concerning the location of the marked SP-like immunoreactions described above, the NK-1R immunoreactions were especially observed for myositis areas and areas that were close to these. NK-1R immunoreactions occurred for white blood cells of the inflammatory infiltrates (Figure 3C) and were also detected in nerve fascicles (Figure 4D). NK-1R immunoreactions were also detected in necrotic muscle fibers (Figure 5C) and blood vessel walls in myositis areas (not shown).

##### Immunoexpression of desmin and CD31

It was clearly obvious that the frequently occurring muscle fibers, which based on the appearance after staining for H&E were interpreted to represent necrotic muscle fibers, were indeed desmin-negative (Figure 5A). Normally-appearing muscle fibers were, on the other hand, showing desmin immunoreaction (Figure 5A).

There were numerous blood vessels showing CD31-immunoreaction in the areas composed of loose connective tissue containing numerous white blood cells, i.e. myositis areas (Figure 2A).

##### NaCl group

SP-like and NK-1R immunoreactions were also detected in white blood cells, blood vessel walls and nerve fascicles in the muscle tissue of the NaCl group, but these reactions were of clearly lower degree and frequency than those seen for the C + Th and SP + C + Th groups. A parallel observation was, as commented on above, that the morphological changes were also less pronounced in the NaCl group (c.f. Figure 1A).

### SP concentration

#### Soleus muscle

The SP concentration was significantly higher in the C + Th group compared with the NaCI group (509 pg/mg vs. 240 pg/mg, p < 0.001), and there was also a significantly higher mean value in the C + Th group than in the SP + C + Th group (experimental side) as well (509 pg/mg vs. 351 pg/mg, p < 0.02) and similarly, there was a difference between the C + Th group and the non-experimental side of the SP + C + Th group (509 pg/mg vs. 295 pg/mg, p = 0.001). There was a tendency of an increase in SP concentration in the SP + C + Th group (concerning both experimental and non-experimental sides) as compared with the NaCI group. The tendency was most pronounced for the experimental side. Nevertheless, the difference was not statistically significant. No significant difference was found between experimental and non-experimental sides for the SP + C + Th group (p = 0.178). All values are presented in Table 1.

#### Gastronemius muscle

The SP concentration was significantly higher in the C + Th group as compared with the NaCI group (531 pg/mg vs. 353 pg/mg, p = 0.017, respectively) (Table 1). It was also significantly higher in the SP + C + Th group (experimental side) as compared with the NaCI group (511 pg/mg vs. 353 pg/mg, p = 0.039, respectively). No significant difference was found between experimental and non-experimental sides for the SP + C + Th group (p = 0.316).

## Discussion

The study shows that the local injections with NEP and ACE inhibitors provoked the myositis/muscle derangement processes that occur in response to the overuse. It was evident that the muscles of animals that had been given the local inhibitory substances showed a more pronounced tissue derangement than those treated with NaCl. The increase was most clearly seen for the soleus muscle in the C + Th group. The present study also shows that the local injections with C + Th and to some extent also the injections with SP + C + Th led to more marked NK-1R and SP-like immunoreactions in the myositis areas and in the adjacent areas. There were higher SP concentrations in the muscle tissue of these two groups than in that of the NaCl group. It was very obvious that the morphological alterations and the upregulations in expressions of SP/NK-1R were much more noticeable for the C + Th and SP + C + Th groups than for animals subjected to the overuse for 1w using our model and when no injections were given [4-6].

There was a tendency for a more pronounced tissue derangement/inflammation in the C + Th group as compared to the SP + C + Th group. There was a significantly higher SP concentration in the C + Th group compared to the SP + C + Th group concerning the soleus muscle. One explanation to the occurrence of the differences between the two groups can be the fact that exogenously given tachykinin, such as SP, is short-lived, why the major effects we see are those of the ACE and NEP inhibitors. For example, it was previously shown that the plasma extravasation elicited by topical application of SP onto rabbit joint capsule is short-lived, the high extravasation initially seen not being observed 24 h after the experiment [34]. Endogenous SP is also known to be stable in human plasma in vitro whilst added exogenous SP disappears from it rapidly [35,36]. Another explanation to the differences between the C + Th and SP + C + Th groups may be that it can not be excluded that the exogenously given SP to a certain extent leads to a down-regulation of the endogenous SP production.

### Marked SP-like and NK-1R immunoreactions and increased SP concentrations

The use of the polyclonal SP antibody showed that there were immunoreactions in blood vessel walls. Reactions at this location were not seen by the use of the monoclonal SP antibody. The polyclonal SP antibody is directed against all isoforms of the protachykinin 1 precursor, why it is possible that not only SP but also other tachykinins are involved in blood vessel function in the muscle tissue.

The marked SP-like/NK-1R immunoreactions that were seen in myositis areas and areas adjacent to these included immunoreactions for the frequently occurring white blood cells and, as seen by use of the polyclonal antibody, the walls of blood vessels. SP-like/NK-1R immunoreactions occurred within large nerve fascicles and frequent SP-like immunoreactive fine varicose profiles were also seen. The study also shows that the SP concentration was increased due to the inhibitor treatments, especially in the C + Th group.

These observations suggest that the NK-1R was activated and that a boostering of SP occurred due to the local inhibitor injections. The latter suggestion is in accordance with findings by Steyaert and colloborators in their studies on SP treatment for ruptured rat Achilles tendons showing that exogenously administered SP had a booster effect on endogenous SP production [22].

### Possible SP effects

It is well described that local SP treatments can have inflammatory-mediating and destructive effects [10,12,19]. The NK-1R can on the whole have a significant role in the damaging processes during the occurrence of injury and inflammation [37].

Despite the well-known fact that tachykinins have pro-inflammatory effects [10,12], the role for tachykinins like SP in healing effects should also be considered. SP is known to have trophic and healing effects [19,38], including in the initial stages of Achilles tendon healing in the rat [22]. Rats with high residual SP levels after capsaicin-induced neuropathy showed more improvements than those with low levels in the healing of the rat Achilles tendon [39].

It is likely that other substances, in addition to tachykinins, are also involved in the inflammatory and reorganization processes that occur in the muscle tissue. Accordingly, it is reported that although SP can evoke an inflammatory respose in the knee joint, this effect is unlikely to be a function on its own [34]. It should here be emphasized that various peptides in the tachykinin family can bind to the NK-1R [40]. We have also ourselves noted that there is an enhanced expression of TNF-alpha in the myositis process in the model used in this study and it was concluded that tachykinin (SP) can be involved in its upregulation [41]. Further studies will reveal which other substances that are also involved. It should here be emphasized that NEP not only hydrolyzes tachykinins but also enkephalins [24,42] and that ACE degrades not only tachykinins but also bradykinin [43]. ACE is also reported to degrade endothelin-1 and possibly also CGRP [26]. This means that the C + Th treatments not only leads to increased SP levels in the tissue.

To summarize, it is presumable that tachykinins such as SP, as is suggested for certain other situations [44], can have double-edged effects in the processes of myositis/muscle derangement, tachykinins being involved in both pro-inflammatory and reparative processes. In accordance with this concept, we noted in our previous study on NK-1R expression patterns in myositis for animals excercised/electrically stimulated for 3-6 weeks and for which animals no injections were given [6], an occurrence of NK-1R expression in both necrotic and regenerating muscle fibers. Further studies will reveal the relationships between the pro-inflammatory and healing effects.

### Bilateral findings

In the study, muscle samples in the group treated with SP + C + Th were analyzed concerning both the experimental side and the contralateral side. It was found that there were tendencies for more marked alterations of the muscle tissue and an increase in SP concentration for both sides, as compared to the NaCl group, the SP increase being especially obvious for the gastrocnemius muscle. This shows that unilateral exercise/electrical stimulation parallelled by injection of local inflammatory-modulating substances leads to effects also contralaterally. Similarly, we have noted that morphological changes [4] and occurrence of upregulations in tachykinin (SP-like) [5] and NK-1 R [6] expressions are seen bilaterally in response to the prolonged (3-6 weeks) exercise/electrical stimulation protocol without injections being given. Our interpretation has been that neural mechanisms are likely to be involved as the morphological changes (the myositis and muscle derangement) occurred focally and not generally and as influences on the structure of nerve fascicles could be observed. Nevertheless, further electrophysiological and CNS studies are needed in order to prove this.

## Conclusions

Previous studies have shown that there is an inflammatory component in the muscle/tendon affection in response to overuse [45]. In studies on flexor digitorum tendons using a rat model of repetitive strain injury it was shown that there was an exposure-dependent increase in SP immunoexpression in the peritendon tissue [3]. The present study gives new information on the involvement of SP within muscle tissue in a condition of marked muscle overuse.

In conclusion, it is here shown that the local injections of C + Th markedly amplified the structural changes in the muscle tissue and lead to increased SP-like and NK-1R immunoexpressions in the myositis areas and increased SP concentrations in the tissue. To a certain extent, the SP + C + Th treatment led to smilar changes. The changes are likely to be attributed to increased effects of endogenously produced substances out of which tachykinins can be important. It is thus possible that these endogenously produced substances have a previously unrecognized importance in mediating effects on muscle tissue in situations with pronounced overuse. It remains for future studies to reveal if blocking of tachykinin effects via use of NK-1 R blocking substances can be of importance in reducing the tissue effects that marked muscle overuse leads to.

## Competing interests

There are no competing interests.

## Authors’ contributions

SF, RL, CB, PS and JY made the conceiving and design of the animal experiments. RL was responsible for the injection treatments. YS performed the experimental stainings and EIA analyses. YS and SF performed the microscopic analyses. YS, SF, JY and PS evaluated the results. YS and SF drafted the manuscript; all the other authors gave comments on the manuscript. All authors read and approved the final version of the manuscript.

## Pre-publication history

The pre-publication history for this paper can be accessed here:

http://www.biomedcentral.com/1471-2474/15/126/prepub
